# MacroH2A subtypes contribute antagonistically to the transcriptional regulation of the ribosomal cistron during seasonal acclimatization of the carp fish

**DOI:** 10.1186/1756-8935-3-14

**Published:** 2010-07-29

**Authors:** I Araya, G Nardocci, JP Morales, MI Vera, A Molina, M Alvarez

**Affiliations:** 1 Laboratorio de Biología Celular y Molecular, Facultad de Ciencias Biológicas, Universidad Andrés Bello, Avenida República 217, MIFAB, Santiago, Chile

## Abstract

**Background:**

Incorporation of histone variants into chromatin is one of the epigenetic mechanisms used for regulation of gene expression. Macro (m)H2A is a histone variant that has two different subtypes in vertebrates: mH2A1 and mH2A2. It is known that mH2A is associated with gene silencing, but recent studies indicate that these mH2A subtypes could contribute more widely to transcriptional regulation. We have previously demonstrated that the gene-reprogramming response mediates adaptation of the carp fish to its environment, and that ribosomal gene transcription is seasonally regulated in carp. However, there have been few studies investigating how epigenetic mechanisms contribute to environmental adaptation and, in particular, to ribosomal cistron regulation.

**Results:**

In this paper, we report the occurrence of differential incorporation of mH2A subtypes into chromatin during seasonal adaptation in the carp, an event that concurs with opposing transcriptional states. Moreover, we observed that enrichment of mH2A1 in the ribosomal cistron during winter, and conversely, enrichment of mH2A2 during summer. mH2A1 consistently colocalizes with a heterochromatin marker (H3K27me2; histone H3 trimethylated at lysine 27) and mH2A2 with a euchromatin marker (H3K4me3; histone H3 trimethylated at lysine 4). Similar results were found for the L41gene, with enrichment of mH2A in the promoter region.

**Conclusions:**

We have characterized both mH2A subtypes from carp fish, and evaluated their participation in the regulation of the ribosomal cistron. Our findings indicate that differential incorporation of mH2A subtypes into the ribosome could regulate gene expression during the acclimatization process in carp. Our results reveal differential chromatin incorporation of the mH2A subtypes during the environmental adaptation process, correlating wtih antagonistic transcriptional states in the carp ribosomal cistron.

## Background

The structure of chromatin adjusts dynamically depending on the functions of the cell. The basic unit of the chromatin is the nucleosome, which in its core particle consists of 146 bp of DNA wrapped around an octamer of histone proteins (two copies of H2A, H2B, H3 and H4), followed by a linker DNA sequence that is bound by H1 histone [1]. Changes in this highly organized structure are produced through various related epigenetic mechanisms such as DNA methylation, post-translational modifications (PTMs) of histones, ATP-dependent chromatin remodeling, and exchange of 'conventional' histones for other variants [2,3]. Interestingly, epigenetic mechanisms seem to allow an organism to respond to environmental changes by adjusting gene expression [4]. In this context, nucleosome reorganization mediated by epigenetic mechanisms has emerged as a crucial step in gene regulation. In addition, histone variants are associated with transcriptional regulation, not only by allowing changes in the composition of nucleosomes, but also by extending the 'histone code' [5]. Diverse histone variants have been described for four types of histone (H1, H2A, H2B and H3), and their biological functions have been characterized. H2A has the largest number of variants, including H2A.X, H2A.Z, H2ABbd and macro(m)H2A, all of which are involved in various cellular functions, including transcriptional regulation, DNA repair, chromosome assembly and developmental segregation [6-9]. Within this family, mH2A stands out by virtue of its unusual size, which is three times that of canonical H2A, and includes a large C-terminal domain (macro domain or non-histone region; NHR) that comprises almost two-thirds of the protein [10], and an N-terminal region enclosing the histone domain [11]. Whereas repetitive genes clusters arrange in tandem encode conventional histones, non-repetitive genes encode histone variants [6]. In mammals, two non-allelic genes encode for the mH2A1 and mH2A2 subtypes. The mH2A1 subtype has two alternatively spliced variants, mH2A1.1 and mH2A1.2, which differ in a short region located at the NHR. In terms of biological function, mH2A is associated with gene silencing. However, recent studies indicate that mH2A subtypes could play additional functions in gene transcription [11-14]. Moreover, the expression pattern of the mH2A subtypes changes during differentiation of various cell types and tissues, and at some stages of embryonic development [8,15,16]. These traits suggest that the role of mH2A is not restricted only to transcriptional repression, but rather acts in the fine control of regulation of gene expression.

Control of gene expression is more complex in organisms that are forced to generate a molecular response against environmental changes [17]. Fish are confronted with large fluctuations in habitat conditions (for example, water temperature, salinity, oxygen concentration, nutrient availability), which compel physiological adaptation of cells and tissues [18]. The close association between the physiology of a fish and its environment makes this organism an excellent model for studying modulation of gene expression by habitat. *Cyprinus carpio *(carp) is a eurythermal fish that has been able to adapt successfully to the most wide-ranging habitats. It is well established that molecular adaptation of carp to environmental changes is coordinated through accurate regulation of gene expression [19,20]. A notable example of this process is ribosomal biogenesis, which is finely modulated during acclimatization, and is directly correlated with season-dependent organization of the nucleolar components [21].

We have previously reported that some epigenetic mechanisms participate in gene regulation during carp acclimatization. A hypermethylated state in the promoter region of ribosomal genes was observed in carp adapted to winter conditions, and mH2A is seasonally regulated during carp adaptation [22]. In this study, we set out to evaluate the role of mH2A in the transcriptional regulation of ribosomal genes, by analyzing separately the involvement of the two subtypes, mH2A1 and mH2A2.

We found that mH2A associates with ribosomal genes during acclimatization, and more importantly, that there is differential enrichment of the mH2A variant subtypes, depending on the seasonal transcriptional activity. These data suggest that these subtypes have different functions, and may operate as opposing epigenetic regulators for transcriptional regulation of ribosomal genes. The seasonal differential enrichment of mH2A1 and mH2A2 consistently correlates with heterochromatin and euchromatin markers, respectively.

## Results

### Determination of cDNA sequences of the carp mH2A subtypes

**Figure 1:**
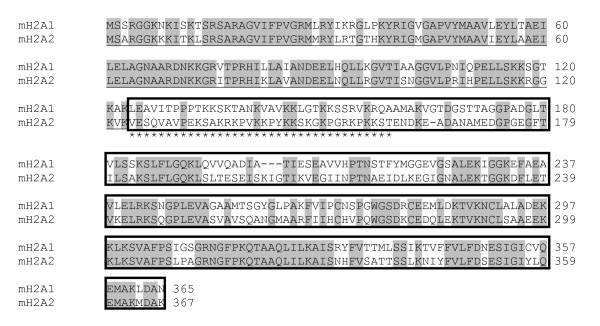
**Amino acid sequence alignment of carp mH2A subtypes**. Carp cDNA sequences of mH2A1 and mH2A2 were aligned using ClustalW software. The histone domain of the protein is underlined, and the macro domain is highlighted within a box. Conserved residues are shown in grey, and the linker region is indicated with asterisks.

We isolated two cDNA sequences encoding for histone variants mH2A1 and mH2A2 from carp [Genbank accession numbers GU585908 and GU585909]. The deduced amino acid sequences show that mH2A1 and mH2A2 respectively contain 365 and 367 residues. These two subtypes share 61% identity across the sequence, but there is strong conservation in the histone domain (79%) and greater divergence in the macro domain (51%). It is noteworthy that in the N-terminal region of the macro domain, which corresponds to the linker region, divergence between these subtypes is extremely high, with only 19% identity (Figure 1).

### Distribution of mH2A subtypes throughout the ribosomal cistron in the carp

**Figure 2:**
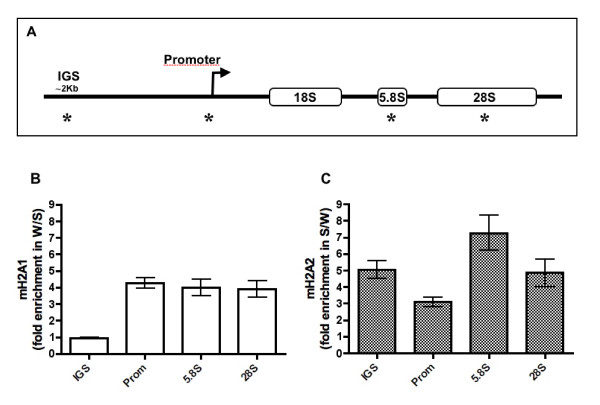
**Assessment of the incorporation of mH2A1 and mH2A2 in the carp ribosomal cistron through chromatin immunoprecipitation (ChIP) assays**. **(A) **Schematic representation of the carp ribosomal cistron. The regions evaluated are indicated by asterisks. Fold enrichment of **(B) **mH2A1 and **(C) **mH2A2 in different regions of the carp ribosomal cistron is represented as the incorporation ratio between winter and summer.

Using chromatin immunoprecipitation (ChIP) and subsequent quantification by real time quantitative (q)PCR, we evaluated the incorporation of the mH2A1 and mH2A2 subtypes along the carp ribosomal cistron (Figure 2). The results showed that enrichment of mH2A1 increased several-fold along the carp ribosomal cistron during winter. In particular, the enrichment was higher (fourfold) in the promoter, 5.8S and 28S regions (Figure 2B). By contrast, the amount of mH2A2 was more abundant during the summer season (Figure 2C), increasing threefold in the promoter region, whereas in the IGS, 5,8S and 28S regions, the increase ranged from fivefold to sevenfold.

### Positioning of mH2A1 and mH2A2 in promoters of genes transcribed by RNA polymerase II during the carp acclimatization process

**Figure 3:**
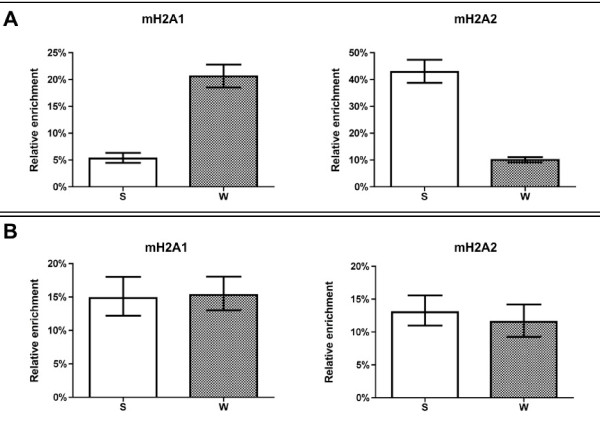
**Evaluation of mH2A1 and mH2A2 enrichment in the promoter regions of the L41 and prolactin genes om seasonal acclimatized carp**. The bars show the relative enrichment of mH2A1 and mH2A2 in the **(A) **the L41 gene promoter region (-1010 to -819 bp) and **(B) **the prolactin gene promoter region (-93 to 13 bp) (n = 3 for both) under both adaptive conditions (summer (S) and winter (W). Standard deviations (± SD) are shown.

In addition to studying the ribosomal distribution in genes, we evaluated the association of the mH2A subtypes with the promoter regions of two genes that exhibit seasonal dependent expression in the carp: the ribosomal protein L41 and prolactin carp genes. Interestingly, and consistent with the results obtained about the level of mH2A in the ribosomal gene analyzed, the amount of mH2A1 was four times greater in the promoter region of the L41 gene during winter compared with summer. By contrast, the amount of mH2A2 was about four times greater in the L41 promoter region in summer than in winter (Figure 3A). Similarly, the seasonal occurrence of these two mH2A variants was evaluated in the promoter region of the prolactin gene using chromatin isolated from pituitary samples of seasonally adapted carp. In contrast to the L41 results, there was no significant variation in the content of mH2A1 and mH2A2 during the winter and summer adaptation periods (Figure 3B).

### Correlation between the enrichment of mH2A subtypes and epigenetic chromatin markers

To investigate whether the differential seasonal enrichment observed for mH2A subtypes during carp acclimatization correlated with either the activated or the repressed transcriptional state, we carried out sequential ChIP assays to assess the colocalization of mH2A1 and mH2A2 with histone post-translational modifications such as H3K4me3 (histone H3 trimethylated at lysine 4) and H3K27me2 (histone H3 dimethylated at lysine 27), which are classic epigenetic markers for activated and repressed transcriptional regions, respectively [23].

**Figure 4:**
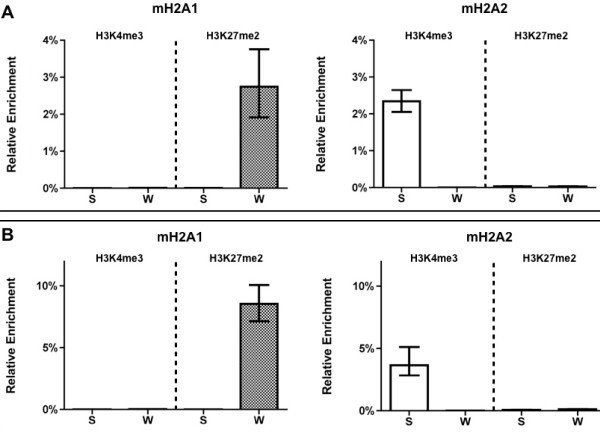
**Colocalization of mH2A1 and mH2A2 with epigenetic markers in carp ribosomal cistron and L41 gene promoters by sequential chromatin immunoprecipitation (ChIP-reChIP) assays**. Colocalization of mH2A1 and mH2A2 in the promoter region of **(A) **the carp ribosomal cistron and **(B) **the L41 gene with the heterochromatin marker H3K27me2 and the euchromatin marker H3K4me3 in summer (S) and winter (W). Standard deviations are shown in each bar.

We evaluated the colocalization of mH2A subtypes and epigenetic markers around the basal promoter region of the ribosomal gene. Our results showed that in winter, mH2A1 colocalized with the H3K27me2 heterocromatin marker, whereas in summer no significant colocalization was observed. By contrast, mH2A2 was colocalized with the H3K4me3 euchromatin marker during summer, whereas in winter no significant colocalization was detected (Figure 4A).

Additionally, we analyzed the epigenetic context in which the mH2A subtypes are incorporated into the L41 promoter region. Again, the activation marker colocalized with mH2A2 and was more abundant in summer, whereas the repression marker colocalized with mH2A1, and only during the winter season (Figure 4B).

## Discussion

Previously, we reported that ribosomal transcription is tightly regulated as part of the compensatory response during seasonal acclimatization of carp [24], involving epigenetic mechanisms such as methylation, post-translational modifications of histone modifications and replacement of conventional histones by their variants [22]. Interestingly, ribosomal transcriptional regulation also correlates with profound rearrangements of the nucleolar components [21]. The present study has increased our understanding of how epigenetic mechanisms mediated by the incorporation of histone variants participate in the adaptive response of an organism to changes in its environment. Moreover, our results unexpectedly suggest that mH2A subtypes are able to contribute independently to transcriptional regulation.

The histone variant mH2A is associated with transcriptional repression, but new evidence seems to indicate that the different mH2A subtypes do not behave in the same way, and consequently may perform different functions. In mouse liver chromatin, larger amounts of mH2A1 are found mainly in silenced genes, but in some cases, larger amounts of mH2A1 have been found in upstream regions of genes with high levels of transcription [25]. Moreover, in human cells, a considerable fraction of mH2A1-containing genes are able to escape repression [14]. Similar studies on mH2A2 are not available. Furthermore, it has been reported that the developmental and tissue expression patterns of the mH2A subtypes differ, supporting the idea that different subtypes have dissimilar functions. For instance, the mH2A1.1 and mH2A1.2 variants change their expression when cell differentiation is induced [15]. More recently, it was reported that mH2A subtypes appear to be differentially expressed during zebrafish embryogenesis [8]. Thus, in the early stages of zebrafish development, expression of mH2A2 is higher than that of mH2A1; the latter begins to be detectable only after 72 hour post-fertilization. mH2A2 was reported to play an important role as an epigenetic regulator of developmental genes [8]. In addition, mH2A1 and mH2A2 both have a tissue-specific expression pattern in adult mouse liver and kidney sections [16]. In summary, the evidence suggests a more complex function for mH2A, in which each subtype could play a role in the fine regulation of gene expression.

Our results reveal that the mH2A subtypes operate in a diverse and functionally opposing manner within chromatin during environmental adaptation of the carp. Thus, the levels of mH2A1 are higher in the ribosomal cistron during winter, a season in which diminished rRNA transcription has been observed [24]. Conversely, levels of the mH2A2 subtype were higher during summer, correlating with the highest ribosomal transcription activity.

To evaluate if this mechanism is exclusive for RNA polymerase I activity or is a general mechanism, we selected two genes that are transcribed by RNA polymerase II, which are seasonally regulated in the carp [26,27]. Thus, we assessed the participation of the two mH2A subtypes in the chromatin organization of the promoter regions of the genes encoding L41 ribosomal protein and prolactin hormone during seasonal adaptation. Interestingly, in the L41 gene promoter, incorporation of mH2A1 was significantly higher in winter than in summer, whereas the mH2A2 variant was higher in summer in the same region, similar to the results for the ribosomal cistron. By contrast, the results for the prolactin gene showed no significant differences between the seasons for either of the subtypese. This result could indicate that the mH2A subtypes work in a tissue-specific manner. In agreement with these findings, it has been reported that the tissue expression patterns and nuclear distribution of mH2A1.2 and mH2A2 subtypes in the mouse kidney are different [16].

Consistent with the differential chromatin enrichment of the mH2A subtypes, we observed that the post-translational modifications of the histones correlated with a particular active or inactive chromatin structure. Thus, when mH2A1 was enriched in the carp ribosomal cistron, it colocalized with a heterochromatin marker (H3K27me2), which coincides with the transcriptional repression previously reported in winter during carp acclimatization, whereas the mH2A2 enrichment in summer colocalized with a euchromatin marker (H3K4me3), correlating with an increase in rRNA transcription [24]. The same colocalization between these histone epigenetic markers and the mH2A subtypes was consistently observed in the promoter region of the L41 gene, which in carp is upregulated during summer [27]. Thus, these results strengthen the idea that these mH2A subtypes could have antagonistic roles, helping to establish the distinct chromatin structure necessary to reprogram gene expression during carp acclimatization.

Whereas our previous report [22] showed the global mH2A2 content being highest during winter, the present data demonstrate that the incorporation of mH2A2 into chromatin at the ribosomal (r)DNA locus is highest during summer. This implies that the mechanism of incorporation of mH2A2 is independent of global protein content during carp acclimatization.

In human and mouse, mH2A1 and mH2A2 proteins shared about 66% identity, with a high degree of conservation of the histone domain but a significant divergence in the macro domain. In carp mH2A, the percentage identity between both subtypes is lower (61%), with macro domain being particularly divergent (51%). This difference is interesting, and could explain the antagonistic function reported here for the carp mH2A subtypes, as this macro domain contains sequence elements that are associated with the biological function of mH2A. In addition, when comparing carp mH2A1 and mH2A2 sequences, we observed that the N-terminal end of the macro domain is considerably different (19% identity), a feature that could support the idea of different functions for different mH2A subtypes. Interestingly, a basic region known as the linker region [10] is associated with the ability of mH2A to associate with histone deacetylases, affecting the acetylation of nucleosomes containing mH2A [11]. In addition, it has been reported that the macro domain is able to interact and inhibit poly[ADP-ribose] polymerase 1 activity, inducing gene silencing [12]. Another report has suggested that mH2A regulates a balance between transcriptional activation and repression through the chromatin remodeling complexes SWI/SNF and ACF [13]. However, recent studies seem to indicate that the mH2A subtypes could have a wider range of functions. For example, in human cells, there is enrichment of mH2A1 on the inactive X chromosome (Xi), playing a role in the repression of gene expression [28]. Similarly, there is enrichment of mH2A1 in large domains in repressed autosomal chromatin, but surprisingly, this histone can also protect a subset of genes from silencing. Thus, it was observed that when there is enrichment of mH2A1 downstream of the transcription start site region in some genes, these genes escape heterochromatin-associated silencing and become transcriptionally enhanced [14].

In summary, our results provide novel experimental evidence that mH2A subtypes could contribute independently and antagonistically to the delicate regulation of the ribosomal genes during the adaptive response of carp. Moreover, our findings confirm that epigenetic mechanisms play a crucial role in ribosomal RNA transcription.

## Conclusions

Our findings suggest that the differential incorporation of histone variant mH2A subtypes could represent an elegant method to drive the fine regulation necessary to ensure intricate adaptive processes. We provide for the first time evidence that mH2A subtypes can behave as antagonistic epigenetic markers. Further studies should be performed to clarify the molecular mechanisms by which mH2A variants are able to influence chromatin expression.

## Methods

### Animal and tissue preparation

The experimental work on fish described in this paper was approved by the Bioethics Committee of the Universidad Andres Bello, Santiago, Chile.

Male carp weighing 1000 to 2000 g were maintained in seasonal environmental conditions as described previously [22]. Tissues from the liver and pituitary were obtained, washed with phosphate-buffered saline (PBS) pH 7.4, and either processed immediately or stored at -80°C until required.

### Isolation of the mH2A1 and mH2A2 cDNA sequences

The complete coding sequence of mH2A2 was obtained by a rapid amplification of cDNA ends (RACE) method (FirstChoice RLM-RACE kit; Ambion, Austin, TX, USA) with adapter and gene-specific primers deduced from a partial carp mH2A2 sequence [Genbank accession number:DQ173494]. The primers used were macro2F and macro2R (listed in Table 1). mH2A1 was amplified by RT-PCR using carp liver tissue cDNA as template and the primers macro1F and macro1R (Table 1), which were derived from an mH2A1 sequence obtained from *Danio rerio *(Genbank accession number:NM_001040361).

**Table 1:** List of primers used for RACE and ChIP assays

Name	Primer sequence 5'→3'
RACE primers	

Macro1F	ATGTCCAGTCGTGGAGGGAAG

Macro1R	TTAGTTTGCGTCGAGTTTGGC

Macro2F	ATGTCAGCCAGAGGAGGAAAG

Macro2R	TCACTTGGCGTCCATCTTTGC

ChIP primers	

IGSF	CAAGTTCCTGAGCCTCGGC

IGSR	GTGGATGAAAGTTTGACCGG

PromF	CCTCAGGCCGCTGTGGGC

PromR:	GTCTGAGTCTCCCAAGGAAGG

Cc5.8F	GTCGATGAAGAACGCAGCTA

Cc5.8R	GCAAAGTGCGTTCGAAGTGT

Cc28F	GATTCCCTCAGTAGCGGCGA

Cc28R	GCCTGAATTACTGCAGCAATG

CcL41F	GCAAACTCGCCTGACCTTAAC

CcL41R	GCCTGAATTACTGCAGCAATG

CcPlctF	CCTGGAGTGCAAGACTCATTGCAT

CcPlctR	GCAGTCTGATTTCCTCTCTTGAGC

16SF	GGGGTTTACGACCTCGATGTT

16SR	GCTTTAAGTATGGGCCCCCCT

### Nuclear extract and protein quantification

Nuclear extracts were prepared from tissue lysates in H buffer (250 mM sucrose, 3 mM CaCl_2_, 1 mM phenylmethylsulfonyl fluoride (PMSF) in 20 mM Tris pH 7.4), which were separated by centrifugation at 3500 rpm for 5 min at 4°C (rotor HB6; Sorvall, Thermo Scientific, Asheville, NC, USA). The pellet obtained was washed with PBS at least three times. The nuclear fraction was applied to a cushion of 0.8 mol/l sucrose, and separated by centrifugation for 10 min at 2400 rpm (HB6 rotor). Protein quantification was performed using a protein assay kit (DC Universal Protein Assay Kit; Bio-Rad, Hercules, CA, USA), and protein integrity was confirmed by SDS-PAGE.

### ChIP

ChIP was performed essentially according to the protocol provided by the manufacturer (Abcam, Cambridge, MA, USA). We used 3 × 10^6 ^nuclei from liver or pituitary tissues in each assay, from three acclimatized carps respectively. Formaldehyde was added to give a final concentration of 1% in ChIP lysis buffer (50 mM Hepes-KOH pH 7.5, 140 mM NaCl, 1 mM EDTA pH 8.0, 1% Triton X-100, 0.1% sodium deoxycolate, protease inhibitor cocktail) and the mixture incubated for 10 min at room temperature. The samples were placed in a sonicator to yield DNA fragments of sizes ranging from 200 to 700 bp. ChIP assays were performed using polyclonal antibodies mH2A1 and mH2A2 (kind gift of Dr Stefan Dimitrov; ENS-Lyon, France), with 0.25 mg anti-mH2A1 and 0.22 mg anti-mH2A2 used per 5 mg of chromatin. The immunoprecipitated chromatin was reverse cross-linked by incubating overnight with proteinase K at 65°C, and the DNA purified by phenol-chloroform extraction. Finally, the recovered DNA was amplified by qPCR using primers to the IGS, promoter region, 5.8S and 28S regions of the carp rDNA, and the promoter regions of the L41 and prolactin genes (Table 1).

The relative enrichment of the mH2A subtypes in the chromatin was measured according to the Pfaffl equation [29], which integrates an 'input control' value that corresponds to the amplified target on the entire chromatin obtained in each experiment, and to an reference control (carp 16S mitochondrial rRNA). This reference gene, we have previously reported as not being affected by seasonal adaptation [30]. Using this equation, the relative enrichment of the mH2A subtypes in the chromatin for each season was calculated. The fold enrichment corresponds to the ratio of relative enrichment of each histone variant in different regions of the rDNA locus in the different seasons (that is, relative enrichment in winter divided by the relative enrichment in summer).

To check reproducibility, each assay was performed in triplicate and repeated with three independent samples. Results represent the mean (± SD) of three experiments. Statistical analyses were performed using the GraphPad Prism V.4 software.

### Sequential chromatin immunoprecipitation assays

For sequential ChIP (ChIP-reChIP) experiments, 5 mg of chromatin were eluted in 10 mM DTT for 30 min at 37°C. The eluted chromatin was diluted 1 : 10 in dilution buffer (1% Triton X-100, 2 mM EDTA, 150 mM NaCl, 20 mM Tris-HCl pH 8 and protease inhibitor), and the second ChIP step was performed as described for the first. The samples underwent immunoprecipitation using 0.6 mg anti-H3K4me3 or 0.7 mg anti-H3K27me2 (both from Abcam). After extensive washing, the chromatin was eluted, and the purified DNA quantified using real-time PCR.

## Competing interests

The authors declare that they have no competing interests.

## Authors' contributions

IA conducted the ChIP and ChIP-reChIP studies. GN and JPM carried out the cloning of mH2A subtypes. MIV and AM assisted with project design and development and drafted the manuscript. MA conducted project design and direction of research, interpretation of results and preparation of this manuscript. All authors read and approved the final manuscript.
